# Steroid hormones and influence of therapeutic drugs in Chinese postmenopausal rheumatoid arthritis patients

**DOI:** 10.3389/fimmu.2025.1589798

**Published:** 2025-05-12

**Authors:** Ying-ying Zhang, Na Yang, Hua-yong Zhang, Jia-jia Ge, Si-min Yan, Dan Han, Qian-ye Qiu, Wei-hong Ge, Qing Shu

**Affiliations:** ^1^ Department of Pharmacy, Nanjing Drum Tower Hospital, The Affiliated Hospital of Nanjing University Medical School, Nanjing, China; ^2^ Department of Pharmacy, Nanjing Hospital of Chinese Medicine Affiliated to Nanjing University of Chinese Medicine, Nanjing, China; ^3^ Nanjing Medical Center for Clinical Pharmacy, Nanjing Drum Tower Hospital, the Affiliated Hospital of Nanjing University Medical School, Nanjing, China; ^4^ Department of Rheumatology and Immunology, Nanjing Drum Tower Hospital, The Affiliated Hospital of Nanjing University Medical School, Nanjing, China

**Keywords:** rheumatoid arthritis, steroid hormones, methotrexate, glucocorticoids, postmenopausal women

## Abstract

**Objective:**

To investigate steroid hormone profiles and therapeutic modulation in Chinese postmenopausal rheumatoid arthritis (RA) patients.

**Methods:**

This cross-sectional study enrolled 138 postmenopausal women, including 88 RA patients stratified by treatment status (23 treatment-naïve, 35 on methotrexate [MTX] monotherapy, and 30 receiving MTX plus glucocorticoids [GC]) and 50 age-matched healthy controls. Using liquid chromatography-tandem mass spectrometry (LC-MS/MS), we quantified 36 steroid hormones/metabolites to assess treatment-associated endocrine alterations. Group comparisons employed non-parametric Kruskal-Wallis test for multi-group comparisons, with *post-hoc* Mann-Whitney U tests and false discovery rate (FDR) correction for multiple comparisons. Statistical significance was defined as p<0.05 after FDR correction.

**Results:**

Untreated RA patients demonstrated significant global steroid dysregulation, characterized by marked suppression of multiple adrenal steroids (including aldosterone, cortisol, and testosterone) compared to healthy controls (all FDR<0.05). This was accompanied by profound alterations in estrogen metabolism, notably a hyperactivated 2-hydroxylation pathway and depleted 16-hydroxylation metabolites (FDR<0.001). MTX treatment partially restored steroid homeostasis, significantly improving aldosterone and androgen profiles (FDR<0.05) toward levels observed in healthy controls. However, the addition of GC therapy further disrupted endocrine balance, significantly suppressing cortisol, testosterone, and total estrogens (FDR<0.05), while pathologically amplifying the 4-hydroxylation pathway (FDR<0.001), a process potentially linked to synovial inflammation.

**Conclusions:**

This study demonstrates that impaired steroidogenesis and estrogen pathway dysregulation are characteristic features of postmenopausal RA, with MTX showing unexpected hormone-restorative effects. While GC therapy provides symptomatic relief, it paradoxically exacerbates endocrine disruption, suggesting the need for personalized hormonal monitoring in long-term GC-treated patients.

## 1 Introduction

Rheumatoid arthritis (RA) is a chronic autoimmune disease affecting approximately 1% of the global population, with a striking female predominance (female-to-male ratio >4:1) (1). Peak incidence occurs around menopause, while pregnancy and oral contraceptive use appear protective, suggesting crucial hormonal involvement in RA pathogenesis (1–3).

**Figure 1:**
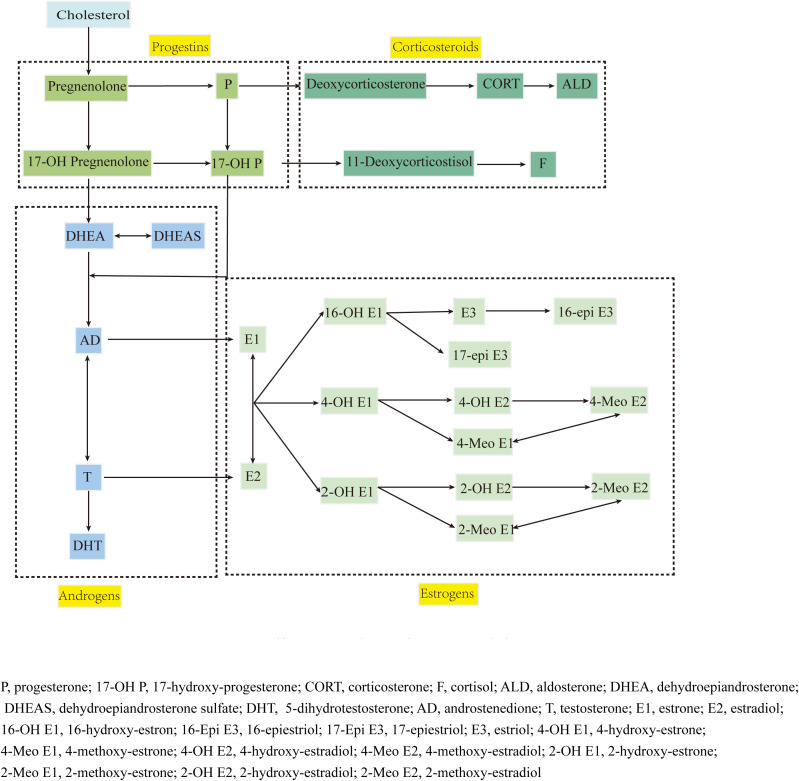
Steroid hormone synthesis and estrogen metabolism.

Steroid hormones, including corticosteroids, progestins, androgens, estrogens and estrogen metabolites (EM), are synthesized through common pathways and regulated by the hypothalamic-pituitary-adrenal (HPA) axis (**Figure 1**), playing crucial yet incompletely understood roles in RA pathogenesis (4). While androgens and progestins generally suppress immune dysfunction (5, 6), estrogen effects vary with concentration and receptor expression patterns (7). The estrogen metabolic landscape is particularly complex, with hydroxylated metabolites showing distinct and sometimes opposing biological activities (7–10).

Standard RA treatment typically combines methotrexate (MTX) with glucocorticoids (GC), achieving remission in 25% of early RA cases (11). While GCs are known to reduce androgen and estradiol levels (12), MTX’s effects on steroid metabolism are unknown. This knowledge gap is particularly relevant for postmenopausal women, who rely primarily on adrenal steroid production.

Therefore, using sensitive liquid chromatography-tandem mass spectrometry (LC-MS/MS) methodology, we conducted the first comprehensive analysis of 36 steroid hormones and their metabolites in Chinese postmenopausal RA patients, examining both disease-related alterations and treatment effects. This study aims to elucidate the complex interplay between RA, steroid metabolism, and commonly used therapeutics in this high-risk population.

## 2 Materials and methods

This cross-sectional study was conducted at Nanjing Drum Tower Hospital from January 2019 to December 2021. Following approval from the hospital’s Ethics Committee (approval number: 2021-246-02) and in accordance with the Declaration of Helsinki principles, we enrolled postmenopausal female RA patients (aged 48–65 years) who met our inclusion criteria: (1) RA diagnosis confirmed by ACR/EULAR 2010 criteria; (2) Either treatment-naïve or on stable treatment regimens for ≥3 consecutive months; (3) Postmenopausal status with FSH >40 IU/L.

Participants were excluded if they had: endocrine disorders (uterine/ovarian diseases, thyroid/adrenal disorders, hypopituitarism), significant comorbidities (any history of malignancy, hepatic/renal insufficiency, diabetes, hypertension, hyperlipidemia), or concurrent medications (hormone replacement therapy, statins, catecholamines, aldosterone synthesis inhibitors, traditional Chinese medicine, biological agents). Fifty age-matched healthy postmenopausal women without medication use served as controls. Sample size was calculated using G*Power software (α=0.05, β=0.20, minimum detectable difference=20% in hormone levels), and quality control samples were analyzed every 20 patient samples with coefficient of variation maintained below 15%.

### 2.1 Sample collection and steroid analysis

Blood Sample Collection and Processing: Serum samples (1 mL) were collected from both RA patients (during their established treatment regimens) and control subjects between 8:00-10:00 AM after overnight fasting. All samples were immediately processed following standardized protocols and stored at -80°C until analysis. Quality control samples were analyzed every 20 patient samples with coefficient of variation maintained below 15%. The analysis was performed using an UPLC I-Class system coupled to a Xevo TQ-S mass spectrometer (Waters Corporation, USA).

Steroid Extraction and Analysis: Serum samples were subjected to liquid–liquid extraction with methyl tert-butyl ether, followed by evaporation to dryness under nitrogen gas and re-dissolution with 100 μl methanol. Then, the dried estrogen samples were derivatized by using dansyl chloride. Finally, quantitative analysis was performed by LC-MS/MS after centrifugation. With reference to the method of Xu et al. (13, 14). Since serum estrogen and EM are generally present as glucuronide and sulfate conjugates, an initial hydrolysis step was performed using beta-glucuronidase/sulfatase to free conjugated estrogen. Therefore, the estrogens measured in this study included free (unconjugated) and total (conjugated + unconjugated) estrogen and EM (13).

Target analytes: 36 steroid hormones (detailed in **Table 1**, pathway shown in **Figure 1**).

**Table 1:** Types of steroid hormones determined.

Non-Estrogen Steroids				
Corticosteroids	Aldosterone (ALD), Corticosterone (CORT), Cortisol (F)			
Progestins	Progesterone (P), 17-hydroxyprogesterone (17-OH P)			
Androgens	dehydroepiandrosterone (DHEA), dehydroepiandrosterone sulfonate (DHEAS), androstenedione (AD), testosterone (T), 5-dihydrotestosterone (DHT)			
Estrogen Metabolites				
Parent estrogens	Total/Free E1 (Estrone)		Total/Free E2 (Estradiol)	
2-hydroxylation pathway	Total/ Free 2-OH E1	Total/ Free 2-OH E2	Total/ Free 2-Meo E1	Total/ Free 2Meo E2
4-hydroxylation pathway	Total/ Free 4-OH E1	Total / Free 4-Meo E1	Total/ Free 4-Meo E2	
16-hydroxylationpathway	Total/ Free 16-OH E1	Total/ Free E3 (Estriol)	Total/ Free 16-Epi E3	Total/ Free 16-Epi E3

Estrogen measurements include both free (before enzymatic hydrolysis) and total (after β-glucuronidase/sulfatase hydrolysis) forms.; Meo, methoxy; OH, hydroxy; Epi, epimer.

Limits of quantification: Corticosteroids, progestins, and androgens: 0.025-1.0 ng/mL; Estrogens and estrogen metabolites: 1.0 pg/mL

### 2.2 Pharmaceutical agents and chemical reagents

The following pharmaceutical agents were used in this study: (1) Methotrexate (MTX): Manufactured by Shanghai Xinyi Pharmaceutical Co., Ltd. (Shanghai, China); (2) Prednisone acetate (GC): Manufactured by Zhejiang Xianju Pharmaceutical Group Co., Ltd. (Zhejiang, China)

### 2.3 Statistical analysis

Statistical analyses were performed using SPSS software (version 23, IBM Corp., Armonk, NY, USA), and figures were generated using Adobe Illustrator 2023. The Kolmogorov-Smirnov test was used to assess the normality of the data distribution. For missing values (<5% of the total data), multiple imputation with 5 iterations was performed. Normally distributed data are presented as mean ± standard deviation (SD), while non-normally distributed data are presented as median with interquartile range.

Our primary statistical methods included analysis of variance (ANOVA) or the Kruskal-Wallis test, complemented by *post-hoc* analyses. In the case of steroid hormone indicators exhibiting non-normal distribution, we employed the non-parametric Kruskal-Wallis test to compare group differences. Subsequently, for significant Kruskal-Wallis test outcomes (p<0.05), we conducted pairwise comparisons using the Mann-Whitney U test with false discovery rate (FDR) adjustment. In instances where the Kruskal-Wallis test was not significant, nominal p-values from individual comparisons were not considered statistically significant, even if they reached statistical significance. The statistical significance threshold was set at FDR<0.05.

## 3 Result

### 3.1 Baseline characteristics

This study enrolled a total of 88 patients with RA, who were categorized into treatment and non-treatment groups: Treatment Groups (n = 65): MTX monotherapy group (RA-MTX; n = 35); Combined therapy group (RA-MTX+GC; n = 30): MTX plus GC. Untreated RA patients (RA-Untreated; n = 23). All treated patients received therapy for > 3 months. MTX dosage: 10-12.5 mg/week; GC dosage: ≤ 15 mg/day prednisone equivalent. Neither the RA-MTX group nor the RA-Untreated group received GC therapy. No significant differences in baseline characteristics were observed between the RA groups and control group (detailed data presented in **Table 2**).

**Table 2:** Demographics and clinical characteristics of study participants.

Characteristics	Control group (n=50)	RA-Untreated (n=23)	RA-MTX (n=35)	RA-MTX+GC (n=30)	*P*-value
Age, years	56.76±4.27	55.21±4.49	55.72±4.45	55.21±4.49	>0.23
BMI,kg/m^2^	22.69±2.24	21.74±2.05	21.77±2.65	23.17±3.83	>0.33
Disease duration, years	–	6.38±6.74	5.11±5.10	5.39±6.07	>0.46
ESR	–	20.85±4.63	17.96±11.51	22.12±13.51	>0.16

RA, rheumatoid arthritis; BMI, body mass index; ESR, erythrocyte sedimentation rate; MTX, methotrexate; GC, glucocorticoids.

Values are presented as mean ± standard deviation (SD). The P-values represent the smallest P-value obtained from between-group comparisons. RA patients were subdivided into three treatment groups: untreated (n=23), MTX (n=35), and MTX plus GC (MTX+GC) (n=30). All between-group comparisons showed no significant differences (P > 0.05).

### 3.2 Levels of steroid hormones

All enrolled patients (n = 88) maintained their prescribed treatment regimens throughout the study period. No intercurrent illnesses were documented in the control group during the blood sampling phase. Among the 36 steroid hormones quantitatively determined, corticosteroids, androgens and progestins were all detected within the range of concentration detection. However, 10 free estrogens (Free E3, Free 2-OH E1, Free 2-OH E2, Free 2-MeO E2, Free 4-OH E1, Free 4-MeO E1,Free 4-MeO E2, Free 16-OH E1, Free 16-Epi E3, Free 17-Epi E3) and 4 total estrogens (Total 2-OH E2, Total 2-MeO E2, Total 4-MeO E1, Total 4-MeO E2) concentrations were all below our detection limit. Therefore, 22 steroid hormones met the analytical criteria and were included in the statistical analysis.

### 3.3 Steroid hormone alterations in rheumatoid arthritis and treatment response

Our comprehensive steroidomic analysis revealed significant dysregulation of the steroid hormone network in rheumatoid arthritis. Initial Kruskal-Wallis testing identified several steroid hormones without significant between-group differences (P>0.05), including Total corticosteroids, CORT, 17-OH P, Total E3, and multiple pathway ratios. These variables were excluded from subsequent pairwise significance marking.

Comparative analysis between healthy controls and untreated RA patients demonstrated significant alterations in approximately 64% (14/22) of measured steroid hormones with significant Kruskal-Wallis test results, indicating widespread endocrine disruption in the pathophysiology of RA. Untreated RA patients displayed a distinctive pattern of steroid hormone dysregulation characterized by comprehensive suppression across multiple steroid classes. The androgen axis showed systemic downregulation, with significant reductions in DHEA, DHEAS, T, and DHT. Within the corticosteroid pathway, aldosterone exhibited substantial reduction, while cortisol showed modest reduction that did not remain significant after multiple comparison adjustment.

Of particular immunological significance was the differential modulation observed across estrogen metabolic pathways. While total estrogen concentrations were diminished in untreated RA, we identified pathway-specific alterations characterized by selective suppression of the 16-hydroxylation pathway concurrent with enhancement of the 2-hydroxylation pathway. This metabolic shift was confirmed through significantly altered hydroxylation pathway ratios. The 4-hydroxylation pathway showed significant differences in the Kruskal-Wallis test, with significant reductions in untreated RA versus controls and significant increases following treatment.

Therapeutic intervention significantly modulated the steroid hormone profile in RA patients (**Table 3**). Comparison between untreated and treated RA cohorts revealed substantial normalization of multiple steroid metabolites, with particularly pronounced effects on Total E2 and aldosterone levels. Interestingly, the 2-pathway/4-pathway ratio showed significant reduction in treated versus untreated patients, suggesting potential mechanistic relevance of estrogen metabolism modulation in treatment response. These findings suggest that standard RA therapies may exert part of their immunomodulatory effects through correction of underlying steroid hormone imbalances.

**Table 3:** Quartiles of serum steroid hormones levels in RA and control group.

Steroid hormone determination and statistics	Control group (n=50)	RA-Untreated (n=23)	RA-Treated (n=65)	Change rate (%)	FDR		Change rate (%)	FDR
				①			②	
Corticosteroids (ng/mL)								
Total (ALD+F+CORT)	105.76 (76.15-151.30)	89.48 (81.57-101.06)	90.38 (53.29-135.92)	-15%		N/A	+1%	N/A
ALD	0.08 (0.04-0.11)	0.02 (0.01-0.05)	0.07 (0.04-0.13)	**-83%**		**<0.001**	**+250%**	**<0.001**
F	105.10 (76.16-148.00)	83.33 (56.35-97.79)	88.51 (50.79-130.40)	**-21%**		**0.01**	+6%	0.51
CORT	1.10 (0.83-2.57)	1.37 (0.85-2.95)	1.13 (0.68-1.97)	+25%		N/A	-18%	N/A
Progestins (ng/mL)								
Total (P+17-OH P)	0.27 (0.24-0.33)	0.30 (0.16-0.49)	0.22 (0.12-0.31)	+11%		0.23	-23%	0.07
P	0.13 (0.12-0.16)	0.08 (0.05-0.10)	0.06 (0.05-0.10)	**-38%**		**<0.001**	-25%	0.65
17-OH P	0.14 (0.11-0.16)	0.20 (0.09-0.38)	0.14 (0.07-0.23)	+43%		N/A	-30%	N/A
Androgens (ng/mL)								
Total (DHEA+DHEAS+AD+T+DHT)	949.20 (785.99-1361.93)	473.45 (194.48-753.84)	225.52 (50.49-622.41)	**-50%**		**<0.001**-	52%	0.06
DHEA	1.47 (0.94-2.01)	0.75 (0.22-1.17)	0.55 (0.10-1.11)	**-49%**		**<0.001**-	-27%	0.48
DHEAS	947.50 (782.83-1359.25)	471.70 (193.97-752.18)	224.11 (50-621.07)	**-50%**		**<0.001**-	-52%	0.36
AD	0.63 (0.53-0.81)	0.58 (0.49-0.68)	0.48 (0.19-0.73)	-8%		0.12	-17%	0.19
T	0.16 (0.12-0.19)	0.11 (0.07-0.13)	0.11 (0.06-0.15)	**-31%**		**<0.001**	**-**	0.62
DHT	0.05 (0.04-0.07)	0.03 (0.03-0.03)	0.03 (0.03-0.04)	**-40%**		**<0.001**	**-**	0.58
Estrogens (pg/mL)								
Total (parent estrogens+2-, 4-, 16-hydroxylation pathways)	310.69 (269.38-403.61)	175.51 (119.28-219.50)	173.56 (124.43-216.74)	**-44%**		**<0.001**	-1%	0.82
parent estrogens (FreeE1+Total E1 +Free E2+Total E2)	162.73 (114.64-207.71)	115.08 (73.70-158.10)	84.24 (61.21-107.36)	**-29%**		**<0.01**	-27%	0.87
Free E1	10.17 (7.71-13.03)	11.57 (8.47-16.43)	8.17 (4.44-13.15)	+13%		0.40	**-29%**	**0.03**
Total E1	79.70 (59.39-116.45)	85.92 (45.94-130.95)	29.13 (13.64-51.21)	+8%		0.74	**-66%**	**<0.001**
Free E2	5.33 (4.40-6.49)	1.92 (1.08-2.98)	1.87 (1-3.25)	**-64%**		**<0.001**	-3%	0.79
Total E2	52.69 (40.98-67.07)	4.41 (3.48-6.72)	44.69 (33.00-54.96)	**-92%**		**<0.001**	**+913%**	**<0.001**
2-, 4-, 16-hydroxylation pathways	129.40 (89.45-187.00)	62.20 (35.20-114.28)	72.99 (44.62-110.72)	**-52%**		**<0.001**	+17%	0.37
2-hydroxylation pathway	6.68 (5.08-8.54)	23.01 (8.02-84.34)	24.45 (10.30-40.03)	**+244%**		**<0.001**	+6%	0.40
Total 2-OH E1	1.00 (1.00-1.00)	16.69 (1-48.02)	18.49 (4.65-35.14)	**+1569%**		**<0.001**	+11%	0.92
Free 2MeO E1	2.35 (1.12-4.25)	3.17 (1.54-6.34)	1.10 (1.00-2.20)	+35%		0.19	**-65%**	**0.003**
Total 2MeO E1	4.63 (3.12-7.62)	3.76 (2.31-7.97)	2.98 (1.93-5.08)	-19%		0.61	-21%	0.10
4-hydroxylation pathway	2.11 (1-7.30)	1.00 (1.00-1.00)	10.41 (1.00-17.26)	**-52%**		**0.003**	**+941%**	**<0.001**
Total 4-OH E1	2.11 (1-7.30)	1.00 (1.00-1.00)	10.41 (1.00-17.26)	**-52%**		**0.003**	**+941%**	**<0.001**
16-hydroxylation pathway	108.46 (63.11-162.42)	20.11 (10.98-29.94)	18.58 (10.56-29.38)	**-81%**		**<0.001**	-8%	0.87
Total 16-OH E1	22.21 (10.77-32.00)	11.28 (1.00-17.96)	10.07 (5.24-18.43)	**-49%**		**0.003**	-11%	0.45
Total E3	3.94 (1.00-9.53)	6.33 (1.84-10.30)	2.23 (1.00-6.96)	+61%		N/A	-65%	N/A
Total 16-Epi E3	9.27 (4.58-26.64)	1.00 (1.00-1.16)	4.70 (1.00-6.31)	**-89%**		**<0.001**	**+370%**	**<0.001**
Total 17-Epi E3	50.37 (27.37-67.91)	1.00 (1.00-1.73)	1.00 (1.00-1.00)	**-98%**		**<0.001**	**-**	0.98
Ratio								
16-pathway/4-pathway	20.58 (4.51-9.66)	12.05 (4.84-21.37)	2.55 (0.96-12.10)	-41%		0.25	**-79%**	**0.003**
16-pathway/2-pathway	8.10 (2.96-12.01)	0.54 (0.22-2.32)	0.57 (0.33-1.04)	**-93%**		**<0.001**	+6%	0.67
2-pathway/4-pathway	2.72 (0.73-5.82)	11.33 (5.12-81.22)	1.34 (0.80-3.00)	**+317%**		**<0.001**	**-88%**	**<0.001**
16-pathway/2,4-pathway	5.50 (2.06-9.20)	0.63 (0.21-1.92)	0.46 (0.23-0.71)	**-89%**		**<0.001**	-27%	0.22
parent estrogens/2-, 4-, 16-pathways	1.26 (0.23-2.03)	1.68 (0.72-2.79)	1.16 (0.73-1.76)	+33%		N/A	-31%	N/A
parent estrogens/2-pathway	13.73 (4.68-23.53)	4.58 (1.25-9.51)	2.46 (1.26-5.29)	**-67%**		**<0.001**	-46%	0.08
parent estrogens/4-pathway	35.15 (7.83-120.37)	72.97 (32.22-125.91)	5.23 (3.92-8.33)	+108%		0.23	**-92%**	**<0.001**
parent estrogens/16-pathway	1.35 (0.75-2.05)	4.09 (2.31-7.01)	3.56 (2.22-4.78)	**+203%**		**<0.001**	-13%	0.29

RA, rheumatoid arthritis; FDR, false discovery rate. Values presented as median (interquartile range). Change (%)①: Control vs RA-Untreated; Change (%)②: RA-Untreated vs RA-Treated. "+" indicating increase and "-" indicating decrease. Bolded values indicate significant differences (FDR p < 0.05) only if the overall Kruskal-Wallis test was significant (p < 0.05). For variables with non-significant Kruskal-Wallis results (indicated by "N/A" in the FDR column), the FDR values are not reported despite potential nominal significance in pairwise comparisons. The RA-Treated group (n=65) comprises patients receiving either methotrexate monotherapy (RA-MTX, n=35) or methotrexate plus glucocorticoids (RA-MTX+GC, n=30), representing standard DMARD treatment strategies. For detailed analysis of differential effects between these specific regimens, please refer to **Table 4**.

To further elucidate treatment-specific effects on steroid hormone profiles, we conducted a detailed comparison between MTX monotherapy and MTX+GC combination therapy, as described in the following section.

### 3.4 Differential impact of MTX monotherapy versus MTX+GC combination on steroid hormone profiles in RA patients

Following statistical recommendations from reviewers, our investigation into treatment-specific effects on steroid hormone homeostasis examined 65 rheumatoid arthritis patients stratified into MTX monotherapy (n=35) and MTX+GC combination therapy (n=30) groups, as presented in **Table 4**.

**Table 4:** Quartiles of serum steroid hormones in different treatment regimens of RA.

Steroid hormone determination and statistics	RA-Untreated (n=23)		RA-MTX (n=35)	RA-MTX+GC (n=30)	Change rate (%)	FDR	Change rate (%)	FDR
					③		④	
Corticosteroids (ng/mL)								
Total (ALD+F+CORT)	89.48 (81.57-101.06)		97.48 (81.19-162.18)	70.68 (13.37-112.19)	+9%	0.47	**-27%**	**0.003**
ALD	0.02 (0.01-0.05)		0.06 (0.02-0.11)	0.08 (0.04-0.16)	**+200%**	**0.006**	+33%	0.27
F	83.33 (56.35-97.79)		94.45 (77.10-150.86)	69.58 (12.96-110.20)	+13%	0.07	**-26%**	**0.01**
CORT	1.37 (0.85-2.95)		1.24 (0.81-2.06)	1.00 (0.20-1.91)	-9%	0.58	-19%	0.22
Progestins (ng/mL)								
Total (P+17-OH P)	0.30 (0.16-0.49)		0.23 (0.18-0.29)	0.20 (0.10-0.33)	-23%	N/A	-13%	N/A
P	0.08 (0.05-0.10)		0.07 (0.06-0.11)	0.05 (0.05-0.08)	-13%	N/A	-29%	N/A
17-OH P	0.20 (0.09-0.38)		0.14 (0.10-0.20)	0.12 (0-0.26)	-30%	N/A	-14%	N/A
Androgens (ng/mL)								
Total (DHEA+DHEAS+AD+T+DHT)	473.45 (194.48-753.84)		591.51 (396.26-1032.89)	61.28 (50.31-191.02)	+25%	0.29	**-90%**	**<0.001**
DHEA	0.75 (0.22-1.17)		1.11 (0.55-1.67)	0.16 (0.10-0.47)	+48%	0.09	**-86%**	**<0.001**
DHEAS	471.70 (193.97-752.18)		589.84 (394.50-1031.04)	60.71 (50-190.45)	+25%	0.23	**-90%**	**<0.001**
AD	0.58 (0.49-0.68)		0.60 (0.40-0.75)	0.31 (0.10-0.61)	+3%	0.75	**-48%**	**0.005**
T	0.11 (0.07-0.13)		0.15 (0.10-0.17)	0.06 (0.05-0.11)	**+36%**	**0.002**	**-60%**	**<0.001**
DHT	0.03 (0.03-0.03)		0.04 (0.03-0.06)	0.03 (0.03-0.03)	**+33%**	**<0.001**	**-33%**	**<0.001**
Estrogen (pg/mL)								
Total (parent estrogens+2-, 4-, 16-hydroxylation pathways)	175.51 (119.28-219.50)		209.39 (172.75-306.73)	134.81 (105.83-178.53)	+19%	0.07	**-36%**	**<0.001**
parent estrogens ( (FreeE1+Total E1+Free E2+Total E2))	115.08 (73.70-158.10)		103.10 (71.95-121.32)	76.73 (58.20-92.73)	-10%	0.21	**-26%**	**0.01**
Free E1	11.57 (8.47-16.43)		11.60 (7.73-17.37)	5.40 (2.95-8.48)	–	0.94	**-53%**	**<0.001**
Total E1	85.92 (45.94-130.95)		48.41 (28.64-68.70)	22.80 (8.38-34.75)	**-44%**	**0.02**	**-53%**	**<0.001**
Free E2	1.92 (1.08-2.98)		3.17 (1.65-4.34)	1.00 (1.00-1.99)	+65%	0.07	**-217%**	**0.001**
Total E2	4.41 (3.48-6.72)		42.83 (8.67-57.22)	44.91 (40.29-52.76)	**+871%**	**0.01**	+5%	0.20
2-, 4-, 16-hydroxylation pathways	62.20 (35.20-114.28)		90.41 (55.50-126.16)	56.67 (38.67-93.22)	+45%	0.19	**-37%**	**0.04**
2-hydroxylation pathway	23.01 (8.02-84.34)		35.69 (10.95-46.17)	18.63 (6.96-37.28)	+55%	0.86	**-48%**	**0.05**
Total 2-OH E1	16.69 (1-48.02)		26.46 (5.73-40.92)	14.85 (3.59-33.01)	+59%	N/A	-44%	N/A
Free 2MeO E1	3.17 (1.54-6.34)		2.09 (1.18-3.68)	1.00 (1.00-1.05)	-34%	0.13	**-52%**	**<0.001**
Total 2MeO E1	3.76 (2.31-7.97)		4.61 (2.85-7.09)	2.33 (1.15-3.08)	+23%	0.94	**-49%**	**<0.001**
4-hydroxylation pathway	1.00 (1.00-1.00)		1.00 (1.00-18.24)	11.38 (6.33-16.99)	–	0.07	**+1038%**	**<0.001**
Total 4-OH E1	1.00 (1.00-1.00)		1.00 (1.00-18.24)	11.38 (6.33-16.99)	–	0.07	**+1038%**	**<0.001**
16-hydroxylation pathway	20.11 (10.98-29.94)		28.93 (12.53-35.39)	16.56 (10.39-19.93)	+44%	0.13	**-43%**	**0.006**
Total 16-OH E1	11.28 (1.00-17.96)		12.88 (8.19-19.16)	6.74 (3.85-16.14)	+14%	N/A	-48%	N/A
Total E3	6.33 (1.84-10.30)		5.30 (1.00-11.83)	1.00 (1.00-4.18)	-16%	N/A	-81%	N/A
Total 16-Epi E3	1.00 (1.00-1.16)		4.51 (1.00-7.81)	4.77 (3.27-6.12)	**+351%**	**0.04**	+6%	0.16
Total 17-Epi E3	1.00 (1.00-1.73)		1.00 (1.00-1.00)	1.00 (1.00-1.00)	–	0.97	–	0.99
Ratio								
16-pathway/4-pathway	12.05 (4.84-21.37)		8.06 (3.09-26.77)	1.15 (0.67-1.87)	-33%	0.69	**-86%**	**<0.001**
16-pathway/2-pathway	0.54 (0.22-2.32)		0.48 (0.23-0.74)	0.86 (0.36-1.63)	-11%	0.33	+79%	0.06
2-pathway/4-pathway	11.33 (5.12-81.22)		6.33 (1.25-20.60)	1.35 (0.98-3.00)	-44%	0.10	**-79%**	**0.006**
16-pathway/2,4-pathway	0.63 (0.21-1.92)		0.46 (0.22-0.70)	0.55 (0.26-0.76)	-27%	N/A	+20%	N/A
parent estrogens/2-, 4-, 16-pathways	1.68 (0.72-2.79)		1.13 (0.67-1.89)	1.26 (0.73-1.75)	-31%	N/A	+12%	N/A
parent estrogens/2-pathway	4.58 (1.25-9.51)		2.32 (1.22-5.15)	2.78 (1.47-5.57)	-49%	N/A	+20%	N/A
parent estrogens/4-pathway	72.97 (32.22-125.91)		19.56 (7.25-108.93)	4.90 (3.75-8.09)	-73%	0.21	**-75%**	**<0.001**
parent estrogens/16-pathway	4.09 (2.31-7.01)		3.32 (1.47-6.00)	3.59 (2.23-4.71)	-19%	N/A	+8%	N/A

RA, rheumatoid arthritis; FDR, false discovery rate. Data are presented as median (interquartile range). Change (%)③ represents RA-Untreated vs RA-MTX comparison, while Change (%)④ represents RA-MTX vs RA-MTX+GC comparison, with "+" indicating increase and "-" indicating decrease. Bolded values indicate significant differences (FDR p < 0.05) only if the overall Kruskal-Wallis test was significant (p < 0.05). For variables with non-significant Kruskal-Wallis results (indicated by "N/A" in the FDR column), the FDR values are not reported despite potential nominal significance in pairwise comparisons.

The comprehensive hormonal analysis revealed distinct patterns of endocrine modulation between therapeutic regimens. Kruskal-Wallis tests showed no significant overall differences (P>0.05) for total progestins, progesterone, 17-OH progesterone, total estriol, total 2-OH estrone, total 16-OH estrone, and several metabolic pathway ratios.

MTX monotherapy significantly altered a subset (27%, 6/22) of measured steroid hormones, with notable elevations in aldosterone (200% increase, FDR = 0.006), testosterone (36% increase, FDR = 0.002), dihydrotestosterone (33% increase, FDR < 0.001), and total estradiol, concurrent with reduced total estrone levels (**Table 4**). These findings suggest MTX selectively modulates specific androgen biosynthesis pathways while differentially regulating estrogen metabolism.

In contrast, MTX+GC combination therapy demonstrated more profound and widespread endocrine effects, significantly modifying 59% (13/22) of the steroid hormone panel (**Table 4**). The combination therapy induced comprehensive suppression across multiple steroid classes, with total corticosteroids (27% decrease, FDR = 0.003), cortisol (26% decrease, FDR = 0.01), and androgens (90% decrease, FDR < 0.001) exhibiting particularly marked reductions. Additionally, we observed significant inhibition of multiple estrogen metabolites coupled with notable enhancement of the 4-hydroxylation pathway. This treatment regimen substantially altered key metabolic pathway ratios, specifically reducing both 16-hydroxylation/4-hydroxylation and 2-hydroxylation/4-hydroxylation ratios. These metabolic shifts suggest glucocorticoids may preferentially direct estrogen metabolism toward the potentially genotoxic 4-hydroxylation pathway while broadly suppressing alternative steroidogenic activities.

It is important to note that according to current RA treatment guidelines, glucocorticoid use is recommended only in the initial phase of treatment and for the shortest possible duration due to potential adverse effects. Our findings of exacerbated hormonal dysregulation with MTX+GC therapy provide additional mechanistic insights that may further inform this clinical recommendation. These findings provide important mechanistic insights into the hormonal consequences of commonly prescribed RA treatment regimens and may inform clinical decision-making regarding therapy selection and monitoring.

## 4 Discussion

This study represents the first comprehensive steroid hormone profiling in Chinese postmenopausal RA patients using high-resolution LC-MS/MS analysis. The simultaneous quantification of 22 distinct steroid hormones provides unprecedented insights into disease-related hormonal dysregulation and treatment effects. While previous studies primarily focused on individual hormones, our pathway-based analysis reveals complex interconnections in steroid metabolism.

### 4.1 Inflammatory mediators drive adrenal insufficiency in RA: evidence and mechanisms

Female steroid hormones are primarily secreted by the adrenal glands and ovaries (15). In postmenopausal women, the adrenal glands become the predominant source of steroid hormone synthesis following ovarian atrophy (4). Our analysis revealed significant reductions in multiple steroid hormones in RA patients compared to controls: aldosterone (-83%, FDR<0.001), cortisol (-21%, FDR=0.01), dehydroepiandrosterone (-49%, FDR<0.001), dehydroepiandrosterone sulfate (-50%, FDR<0.001), testosterone (-31%, FDR<0.001), dihydrotestosterone (-40%, FDR<0.001), and total estrogens (-44%, FDR<0.001). Importantly, when we specifically analyzed the untreated RA group (n=23), these hormonal reductions persisted without the confounding effects of medication, with aldosterone (-83%, FDR<0.001), cortisol (-21%, FDR=0.01), DHEAS (-50%, FDR<0.001), testosterone (-31%, FDR<0.001), DHT (-40%, FDR<0.001), and total estrogens (-44%, FDR<0.001) all significantly reduced compared to controls (**Table 3**). These findings strongly suggest intrinsic impairment of adrenal function in postmenopausal RA patients independent of treatment effects.

Previous studies have documented various aspects of adrenal dysfunction in RA patients. Filippa et al. reported glucocorticoid-induced iatrogenic adrenal insufficiency in 48% of RA patients on long-term GC treatment, while adrenocortical dysfunction was also observed in patients without GC treatment (16). Yavropoulou et al. found comparable adrenocorticotropin (ACTH) levels between RA patients and healthy controls, despite significantly lower steroid hormone levels in the RA group (17). Furthermore, Straub et al. demonstrated that the reduced steroid hormone levels were not due to renal clearance or liver metabolism, but rather to decreased adrenal production or enhanced downstream hormone conversion (18).

Inflammatory cytokines significantly influence steroid hormone synthesis and receptor function through multiple mechanisms. Tumor necrosis factor-alpha suppresses steroidogenesis by downregulating steroidogenic enzymes, particularly steroidogenic acute regulatory protein and cytochrome P450 side-chain cleavage enzyme, thereby reducing cortisol production in adrenal cells (19). The regulatory effects of interleukin-1beta and interleukin-6 (IL-6) on the HPA axis have been well documented, with both cytokines stimulating ACTH secretion and subsequent glucocorticoid production (20, 21). Furthermore, pro-inflammatory cytokines modulate sex hormone receptor expression, as demonstrated by the complex interaction between estrogen signaling and inflammatory pathways (22). Understanding these cytokine-mediated effects on steroidogenesis and hormone receptor function provides crucial insights into endocrine dysfunction in inflammatory conditions such as RA, and may help explain the observed adrenal insufficiency in our patient cohort.

### 4.2 Altered estrogen metabolism pathways in rheumatoid arthritis: implications for disease pathogenesis

Following our observation of global steroid hormone alterations, detailed pathway analysis revealed significant changes in estrogen metabolism in RA patients. The most striking finding was the mutual imbalance of hydroxylation pathways: in **Table 4**, both the 4-hydroxylation and 16-hydroxylation pathways showed significant suppression (decreased by 52% and 81%, respectively; FDR<0.001), while the 2-hydroxylation pathway showed a marked elevation (increased by 244%; FDR<0.001). Therefore, the 16-hydroxylation/2-hydroxylation pathway ratio in RA patients was 15-fold lower than in controls (FDR<0.001). The analysis of specific metabolites further supported this model. In the 16-hydroxylation pathway, we observed sequential decreases in key metabolites: Total 16-OH E1 showed approximately a 2-fold reduction (49%, FDR=0.003), Total 16-Epi E3 showed a 9-fold reduction (89%, FDR<0.001), while Total 17-Epi E3 exhibited a 50-fold decrease (98%, FDR<0.001). In contrast, metabolites in the 2-hydroxylation pathway were significantly elevated, with Total 2-OH E1 showing a 16-fold increase (1569%, FDR<0.001).

These alterations parallel findings in postmenopausal osteoporosis patients, where increased serum 2-hydroxylated metabolites (2-OH E1 and 2-MeO E1) and decreased 16-hydroxylated metabolites (16-OH E1 and E3) have been reported (23, 24). This association is particularly relevant as RA patients have twice the incidence of osteoporosis compared to age-matched controls (25).

Importantly, we observed distinct compartment-specific patterns. While serum showed predominant 2-hydroxylation, urinary measurements revealed opposing trends: 2-hydroxylation estrogens were 10-fold lower and the 16-OH E1/2-hydroxylation pathway ratio was 20-fold higher in RA patients (26). Moreover, synovial fluid analysis showed elevated levels of 16-OH E1 and 4-OH E2 (27), suggesting tissue-specific metabolism.

The biological implications of these compartment-specific differences are significant. RA synovial cells predominantly synthesize 16-OH E1, which, together with 16-OH E2, functions as a downstream estrogen metabolite influencing monocyte proliferation. The local predominance of 16-hydroxylation pathway estrogens in synovial tissue may contribute to inflammation and tissue hyperplasia (28). These tissue-specific differences in estrogen metabolite distribution between synovium, serum, and urine suggest complex regulatory mechanisms in RA pathogenesis.

### 4.3 Impact of MTX and glucocorticoid treatment on steroid hormone profiles in rheumatoid arthritis

To better understand medication effects on steroid hormone levels, we analyzed differences between RA-Treated and RA-Untreated groups (**Table 3**). Our quantitative analysis revealed significant variations in steroid hormone concentrations between RA-Untreated and RA-MTX groups, as well as between RA-MTX and RA-MTX+GC groups (**Table 4**).

The effects of MTX on steroid hormones have been poorly characterized, particularly in RA patients. While Grosen et al. reported that MTX therapy did not affect serum T levels in American men with inflammatory bowel disease (29), our study demonstrates distinct hormone-restorative effects in Chinese RA patients. Specifically, MTX treatment normalized multiple abnormally reduced hormones: aldosterone (+200%, FDR=0.006), testosterone (+36%, FDR=0.002), and dihydrotestosterone (+33%, FDR<0.001) (**Table 4**). Additionally, MTX treatment significantly increased total E2 levels (+871%, FDR=0.01), suggesting a potential impact on estrogen metabolism pathways. These population-specific responses suggest that ethnic differences may influence MTX’s endocrine effects, though the underlying mechanisms require further investigation.

In contrast, our research provides compelling evidence that GC treatment disrupts hormone homeostasis when combined with MTX therapy. GC supplementation induced comprehensive hormonal suppression: total androgens (-90%, FDR<0.001), total estrogens (-36%, FDR<0.001), and total corticosteroids (-27%, FDR=0.003) (**Table 4**). These findings are particularly concerning given the high prevalence of GC-related complications in RA treatment. Previous studies reported that 28% of RA patients develop iatrogenic adrenal insufficiency, with over 65% unable to discontinue GC treatment (30). Borresen et al. further documented secondary iatrogenic adrenal insufficiency in 48% of their studied RA patients (31). These adverse effects likely result from GC’s dual mechanism of action: direct suppression of hypothalamic CRH production and ACTH secretion, coupled with indirect effects through IL-6 reduction (32, 33).

### 4.4 Clinical implications and future perspectives

This study provides crucial clinical insights into RA management through hormone profile analysis. It is the first to report the significant hormone-restorative effects of MTX. While GC are a typical treatment for RA, current EULAR guidelines recommend their short-term use (typically ≤3 months) at the disease’s onset, with prompt tapering (34). Although long-term GC therapy can alleviate symptoms, it may exacerbate hormonal metabolic disturbances, as demonstrated in this study, and is significantly linked to adverse effects like osteoporosis and diabetes (32, 34, 35). This underscores the necessity of strictly limiting prolonged GC use, especially in postmenopausal RA patients who exhibit baseline hormonal insufficiency and increased susceptibility to GC-induced endocrine interference. Regular hormone monitoring (e.g., steroid analysis every 6 months) is advised for this population to facilitate early detection of GC-related complications. Based on these findings, we propose a stepped treatment strategy: 1) Prioritize MTX monotherapy for patients with severe hormonal imbalances; 2) When GCs are unavoidable, employ the lowest effective dose alongside hormonal profiling; 3) For persistent hormonal deficiencies, consider targeted replacement therapy (e.g., DHEA supplementation).

Despite these findings, several limitations should be addressed in future studies. First, the single-center design and sample size (n=88) limit result generalizability. Second, the cross-sectional nature precludes assessment of temporal hormone changes. Third, mechanistic understanding of MTX’s hormone-restorative effects remains incomplete. Future multi-center studies should: (1) validate these findings in larger cohorts (suggest sample size >200), (2) conduct longitudinal assessments with standardized time points, and (3) investigate molecular mechanisms through *in vitro* and animal studies.

## 5 Conclusions

This LC-MS/MS study provides novel insights into steroid hormone metabolism in postmenopausal RA patients. Our findings further support that impaired steroid synthesis is closely associated with RA pathogenesis, with significant alterations observed in 64% (14/22) of measured hormones. Notably, we demonstrate for the first time that MTX therapy can help restore abnormally reduced steroid hormones toward normal levels in 27% (6/22) of measurements. Although glucocorticoid treatment clinically improves symptoms, it exacerbates hormonal dysregulation in 59% (13/22) of measurements, particularly by enhancing 4-hydroxylation pathway metabolism while suppressing 2- and 16-hydroxylation pathways.

## Data Availability

The original contributions presented in the study are included in the article/supplementary material. Further inquiries can be directed to the corresponding authors.
